# Petrophysical assessment of reservoir quality and recoverable reserves in fluviodeltaic Nubian sandstone Saqqara field Gulf of Suez Egypt

**DOI:** 10.1038/s41598-025-18424-w

**Published:** 2025-09-12

**Authors:** Abdelbaset M. Abudeif, Ahmed E. Radwan, Mohammed A. Mohammed, Faten A. Tawfik

**Affiliations:** 1 Geology Department, Faculty of Science, Sohag University, Sohag, Egypt; 2 Faculty of Geography and Geology, Institute of Geological Sciences, Jagiellonian University, Krakow, Poland

**Keywords:** Petrophysical evaluation, Shale volume (Vsh), Reservoir quality, Hydrocarbon reserves, Nubia formation, Porosity, Permeability, Recoverable hydrocarbon estimation, Solid Earth sciences, Geophysics

## Abstract

Heterogeneous reservoirs require integrated petrophysical rigor to improve the accuracy of hydrocarbon reserve calculation, minimize uncertainty in reservoir characterization, and optimize development options. Using the Fluvio-Deltaic Nubian Sandstone in the Saqqara Field, Gulf of Suez, as an example, this study uses multi-petrophysical workflows, such as well-log correlation, core-log calibration, petrophysical evaluation, petrophysical distribution maps, and multi-petrophysical cross-plot for lithology, fluid saturation, and reservoir parameters, across four important wells (GS323-1, GS323-2 A, GS323-3, and GS323-4 A). Original hydrocarbons in place (OHIP) and recoverable reserves were calculated using volumetric methods, and thermal maturity was evaluated using geothermal gradient estimations. Lithological interpretation, based on neutron-density cross-plots, confirms the dominance of clean sandstone with minimal clay and anhydrite cementation. Thermal analysis indicates a moderate geothermal gradient (~ 2.75 °C/100 m) and formation temperature (~ 147 °C), supporting hydrocarbon maturation. The reservoir exhibits excellent quality, with low shale volume (~ 0.6%), total porosity averaging 13.5%, and high hydrocarbon saturation (~ 78.5%). Despite variations in permeability, significant net pay zones (ranging from 108.5 to 481.5 ft) offer robust development potential. Using volumetric methods, the estimated hydrocarbons in place reach ~ 106 billion barrels, with ~ 31 × 10^9^ barrels considered recoverable. These findings highlight the strategic value of the Nubian Formation in hydrocarbon planning and underline the critical role of integrated petrophysical analysis in maximizing production efficiency in mature basins. Moreover, the insights derived from this study can be effectively applied to similar sandstone reservoirs worldwide, contributing to improved resource management and energy security.

## Introduction

**Fig. 1:**
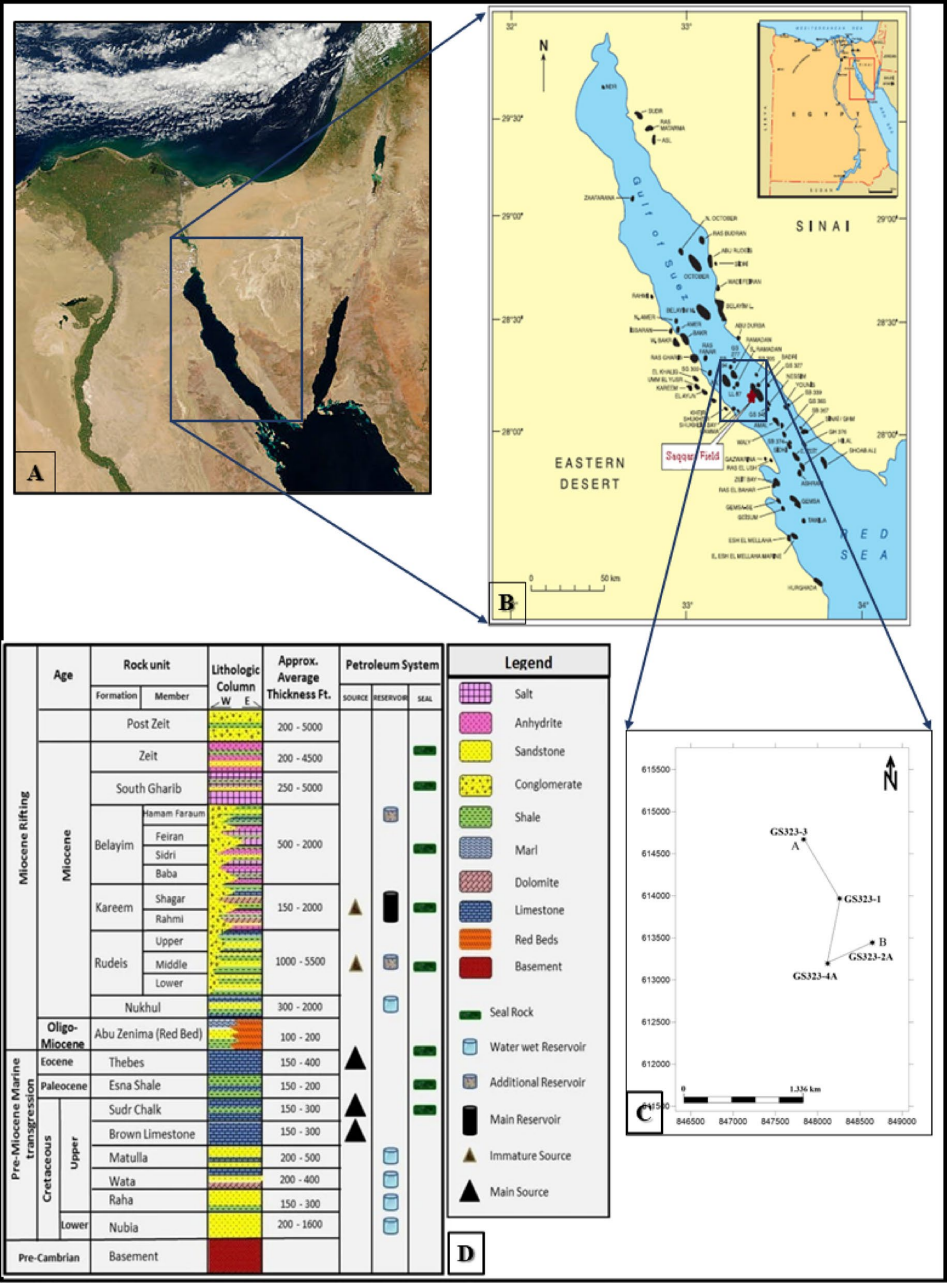
Overview of the Saqqara Field in the south-central Gulf of Suez, Egypt, integrating satellite imagery, geological maps, and stratigraphic sections. (**A**) Landsat image showing the Gulf of Suez’s regional setting^37^. (**b**) Zoomed-in map of the Saqqara Field highlighting major oil fields and tectonic features, modified from^42^. (**C**) Base map detailing well locations (e.g., GS323-1, GS323-2 A, GS323-3, GS323-4 A) after^19^. (**d**) Stratigraphic column of the southern part of the Gulf of Suez, modified after^32^.

The Gulf of Suez (Fig. 1A) is a prominent geological region in Egypt, recognized for its complex tectonic architecture and diverse sedimentary formations, which have established it as an energetic center for petroleum exploration and geological researches^1^. Extending approximately 300 km in length and 30 to 40 km in width, the Gulf aligns parallel to the Red Sea. Over time, it has undergone numerous tectonic events, spanning from the Cretaceous to the Eocene periods, which have led to extensive faulting and fracturing. These geological processes have created distinct structural traps, perfectly suited for hydrocarbon accumulation. As a result, the Gulf of Suez acts as one of Egypt’s most significant and active petroleum-producing regions^1^. Because of its proven hydrocarbon potential, it is a perfect location for sophisticated reservoir characterisation research aimed at optimizing the development of mature fields.

The Saqqara field, the area under study, is located offshore in the southern Gulf of Suez lies approximately 3.5 km west of El Morgan Field, 7.5 km south of Ramadan Oil Field and 3.5 km east of Edfu Oil Field. It is located between longitudes from 33° to 33°50’ E and latitudes from 28° to 28°50’ N (Fig. 1B, C)^2^. The Saqqara Field, discovered in 2003, is one of the significant hydrocarbon discoveries in the Gulf of Suez. Structurally, it is primarily controlled by tilted fault blocks and closures formed by normal faulting. The Nubian Sandstone Formation is the main reservoir in this field and is the focus of the present study. This formation exceeds 2,200 feet in thickness and is subdivided into four main zones (A, B, C, and D). While most zones are dominated by sandstone, Zone B is characterized by dark shale with sparse or barren fauna. The field production has challenges, and the accurate reserves are not yet solved due to the varied petrophysical parameters.

Petrophysical analysis plays a crucial role in characterizing reservoir properties such as porosity, permeability, and fluid saturation, which directly affect hydrocarbon recovery and field performance. This analysis helps delineate reservoir zones, estimate reserves, and optimize field development plans, including well placement and recovery strategies. It also serves as a bridge between geological modeling and reservoir engineering. Understanding reservoir properties not only benefits local field development but also holds broader relevance. On a global scale, accurate reservoir evaluation contributes to improving energy management, increasing extraction efficiency, and supporting the advancement of innovative recovery techniques. These insights are vital for achieving more sustainable and economically viable exploitation of hydrocarbon resources worldwide.

Numerous studies conducted between 2012 and 2024 have significantly enriched our understanding of the Nubian reservoir’s stratigraphy, sedimentology, diagenesis, and structural framework along the Gulf of Suez, however the Saqqara Field have gained less attention compared to other oil fields in this area. These efforts have laid the foundation for more effective exploration and development strategies within the Gulf of Suez. The previous studies relevant to this work can be categorized as follows:

- Geological Studies: Focused on stratigraphy, lithology, and regional geological frameworks, these studies have been conducted by different authors^3–14^.
- Petrophysical Analysis: Addressing the petrophysical properties and well-log interpretation, contributions in this area include those by^15–19^.
- Reservoir Evaluation: Research on reservoir quality, hydrocarbon potential, and productivity has been carried out^20–30^.
- Tectonics and Structural Geology: Studies on structural analysis, fault systems, and the tectonic framework include the work of different authors^6,31^.
- Collectively, these studies have made substantial contributions to refining resource evaluation methodologies and improving reservoir management practices, paving the way for more effective and sustainable hydrocarbon recovery.

While many previous studies^3–31^ have addressed the geology, stratigraphy, and structural attributes of the Nubian Formation along the Gulf of Suez. However, there is currently a lack of research on the integration of contemporary log-based evaluation across numerous wells with an emphasis on calculating hydrocarbon saturation and net pay thickness in Saqqara Field. This study fills that gap by offering a systematic evaluation of reservoir characterization and reserve evaluations unique to the Saqqara Field.

Accordingly, this study aims to deliver a comprehensive petrophysical assessment of the Nubian reservoir in the Saqqara Field through the integration of well log and core data, ultimately enhancing reserve estimation and guiding field development initiatives.This study differentiates itself by employing a multi-disciplinary workflow that combines advanced well log interpretation, shale volume quantification, porosity evaluation, and net pay analysis across four strategically located wells (GS323-1, GS323-2 A, GS323-3, and GS323-4 A), enhanced by supporting core sample reports. These evaluations allow for estimating both the original oil in place and the recoverable reserves. In order to clarify patterns in hydrocarbon saturation and thickness—which are crucial for directing field development strategies—the study also looks into the spatial variability in reservoir metrics. It also has broader implications for reservoir characterization in the Gulf of Suez and other mature basins of a similar nature worldwide.

## Geological setting of the Gulf of Suez

### The stratigraphy of the Gulf of Suez

The stratigraphy of the Gulf of Suez durations from Precambrian to Holocene, and it can be divided into three major sequences: the pre-rift sequence (Pre-Miocene), the syn-rift sequence (Miocene), and the post-rift sequence (Post-Miocene) (Fig. 1D)^32^. The pre-rift sequence consists of the following formations, from bottom to top: the Precambrian basement, Nubian sandstone, the Nezzazat Group (a mixed-facies section), and a thick section of predominantly carbonate Upper Cretaceous chalk, followed by Paleocene shale and Eocene limestones^34^. The syn-rift sequence includes a clastic red bed section of the Abu Zenima Formation (Oligocene - Early Miocene) and Miocene sediments^19^. These Miocene sediments are further divided into two facies: (1) clastic facies, including the Nukhul, Rudeis, and Kareem Formations. (2) evaporite facies, comprising the Belayim, South Gharib, and Zeit Formations^34^. Finally, the post-rift sequence is primarily composed of carbonate and clastic sediments, many of which are considered to be high-quality source and reservoir rocks^33–35^.

The Nubian sandstone formation, which is the focus of this study, is considered one of the most prolific potential reservoirs in the Gulf of Suez, with thicknesses reaching up to 740 feet in the Saqqara field^5^. It is dated as Paleozoic to Lower Cretaceous in age and was deposited in a continental-fluviomarine to marine environment^23^. The formation primarily consists of sandstones with interbedded shales and a few carbonated streaks^35^. It is characterized by high porosity (10–29%) and permeability (70–850 md), with a net pay thickness ranging from 230 to 700 feet. The reservoir quality is primarily influenced by diagenesis, the shaliness of the formation, and burial depth (compaction)^1,5,23,31,36^.

### Structural setting

**Fig. 2:**
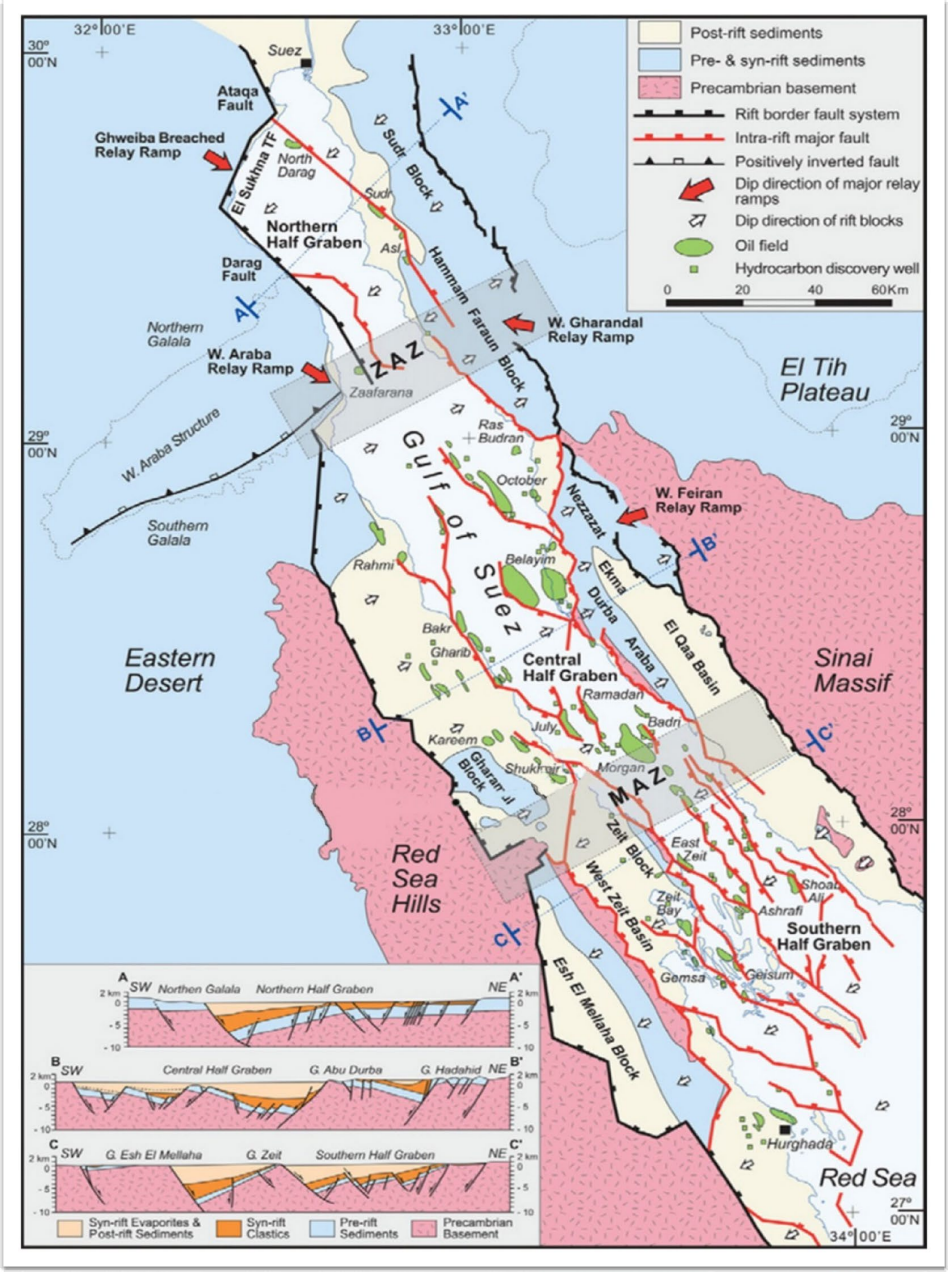
Simplified structural map of the Gulf of Suez rift, highlighting major tectonic structures, fault systems, and offshore fault zones. The map categorizes geological units into post-rift sediments (beige), pre- & syn-rift sediments (gray), and Precambrian basement rocks (pink), illustrating rift evolution and hydrocarbon potential. The rift is divided into three primary tectonic zones: Northern, Central, and Southern Half Grabens. Structural cross-sections (A-A’, B-B’, C-C’) provide insights into faulting, subsidence, and reservoir potential. The map is sourced from^43,44^.

The Gulf of Suez is a rift graben characterized by a highly complex structure, resulting from intense tectonic activity that began during the Oligocene and continued through the post-Miocene^32^. The structural setting of the Gulf of Suez is divided into three provinces: northern, central, and southern, each with distinct fault polarities (Fig. 2). In the northern and southern provinces, the normal faults predominantly dip to the northeast, with strata dipping in the opposite direction. In contrast, the normal faults in the central province commonly dip to the southwest, while the strata dip to the northeast^37^. The northern rift-transverse accommodation zone (Zaafarana accommodation zone) separates the northern and central provinces^38^while the southern rift-transverse accommodation zone (Morgan accommodation zone) separates the central and southern provinces (Fig. 2). The structural features of the Gulf of Suez are predominantly controlled by two main trends: (1) the Clysmic trend, which runs parallel to the Gulf of Suez in a northwest-southeast orientation, and (2) the Aqaba trend, which runs parallel to the Gulf of Aqaba in a northeast-southwest direction. These structural features, along with combination entrapments, are prevalent in the Gulf of Suez^33,36,39–41^. The Gulf of Suez rift is divided into three primary tectonic zones: Northern Half Graben, Central Half Graben, and Southern Half Graben^37^. These segments represent different stages of faulting and subsidence, playing a key role in hydrocarbon accumulation and trap formation. Each of these rift segments contains relay ramps, which are structural transition zones where displacement on a fault system is transferred from one fault segment to another. The following relay ramps are marked: Ghweiba Breached Relay Ramp, W. Gharandal Relay Ramp, W. Feiran Relay Ramp, and W. Araba Relay Ramp^38^. These relay ramps facilitate fault propagation and influence fluid migration pathways within hydrocarbon reservoirs^19^. The map highlights the major rift border fault systems (thick black lines) and intra-rift faults (thin red lines), which accommodate crustal extension in the Gulf of Suez region^19^. The positively inverted faults (dashed red lines) represent areas where previous extensional faults have undergone subsequent compression or uplift, significantly impacting petroleum system dynamics^37^. The oil fields (green shaded areas) and hydrocarbon discovery wells (green circles with black dots) highlight the key locations of oil and gas accumulations^38^. These fields are primarily aligned along the rift’s major fault systems, which create structural traps and enhance reservoir potential. Major oil fields include October Field, Morgan Field, Ramadan Field, Belayim Field, and Ras Sudr Field^43^. These fields are critical for petroleum exploration in the Gulf of Suez and demonstrate the importance of structural control on hydrocarbon entrapment. This structural map of the Gulf of Suez rift provides a comprehensive overview of the region’s fault systems, sedimentary sequences, and hydrocarbon potential^42^. The identified rift segments, relay ramps, and tilted fault blocks play a crucial role in controlling the distribution of oil fields and reservoir quality^43^.

## Materials and methods

**Fig. 3:**
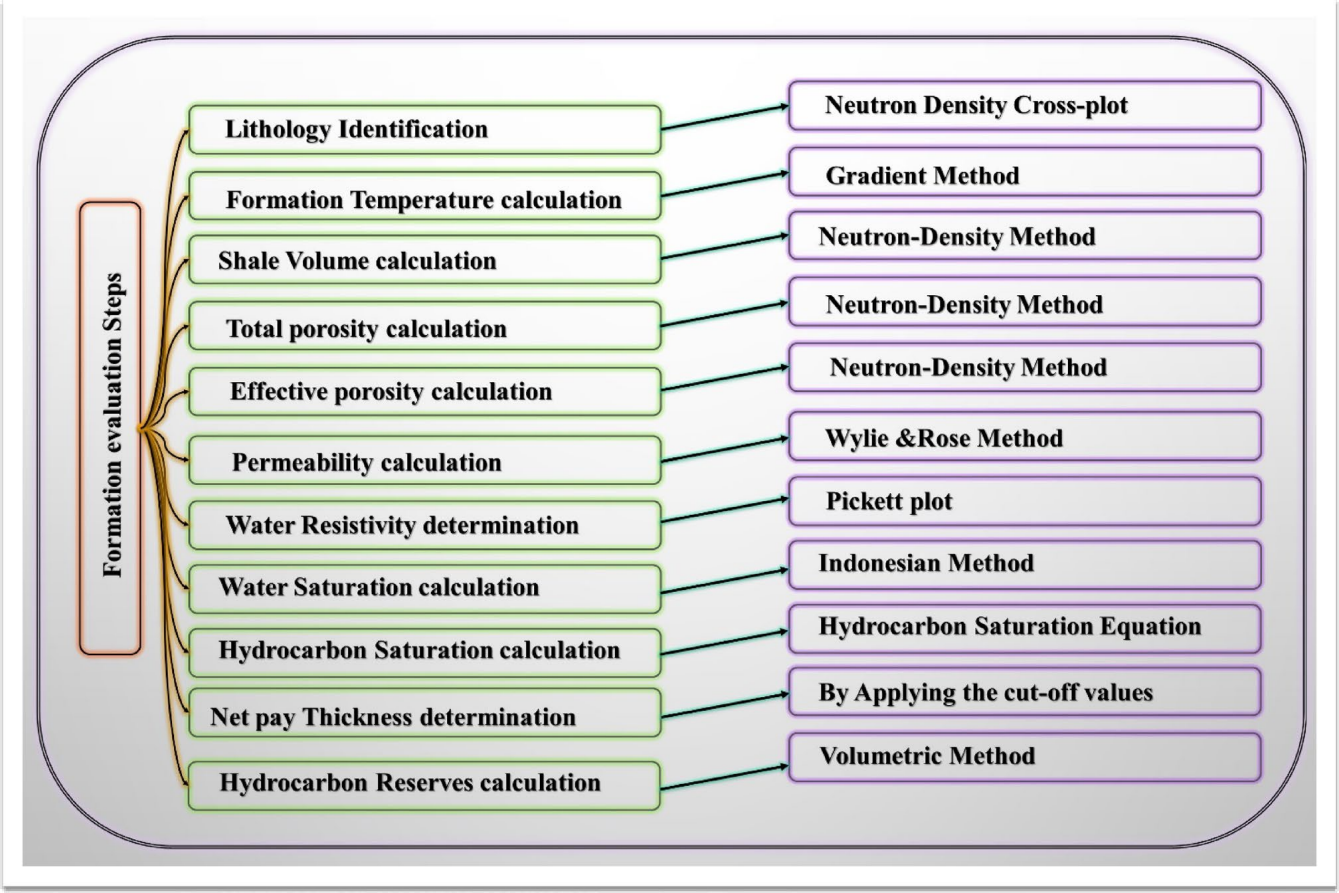
Flowchart illustrates the formation evaluation steps used in the study. The chart outlines key petrophysical calculations, including lithology identification, porosity, permeability, water and hydrocarbon saturation, and hydrocarbon reserves estimation. Each step is associated with specific methods such as the Neutron Density Cross-plot, Wyllie & Rose Method, Pickett Plot, and the Volumetric Method, providing a systematic approach to reservoir characterization and hydrocarbon assessment.

The dataset utilized in this study consists of a comprehensive suite of open-hole logs, including caliper, gamma ray, deep resistivity, density, neutron, sonic, and photoelectric (PEF) logs, obtained from four boreholes in the Saqqara Field (GS323-1, GS323-2 A, GS323-3, and GS323-4 A). Additionally, the dataset incorporates core analysis reports and subsurface geological studies provided by the Gulf of Suez Petroleum Company (GUPCO). The well-log analysis was conducted using equations and cross-plots to assess the Nubia Formation reservoir, with petrophysical parameters determined through the application of Techlog software. The workflow for formation evaluation is summarized in the flowchart (Fig. 3), followed by a detailed description of each step in the evaluation process.

### Lithology identification (Via the Neutron-Density cross-plot)

The neutron-density cross-plot was employed to identify the main reservoir rock types, such as quartz sandstone, limestone (calcite), and dolomite, as well as shale and certain evaporites. This cross-plot relies on the differences in neutron porosity (ΦN) and bulk density (ρb) measurements, which allow for the distinction of these rocks based on their unique physical properties. Additionally, the cross-plot proved effective in detecting the presence of gases. Gas-bearing zones typically exhibit a decrease in neutron porosity readings and an increase in bulk density due to the lower electron density of gases compared to liquids or solid rocks. This makes gas zones easily distinguishable on the plot^45^. In the neutron-density cross-plot the X-axis (Horizontal Axis) represents the bulk density (ρb), which is measured in g/cm³ and the Y-axis (Vertical Axis) represents neutron porosity (ΦN), usually measured in porosity units (p.u.) or as a percentage^45^.

### Formation temperature calculation

The estimation of the temperature of the formation helps to evaluate the maturity of organic matter and whether hydrocarbons are in the oil or gas phase, also the excessive temperatures may lead to hydrocarbon cracking into gas rather than oil. Temperature affects fluid viscosity and density, improving flowability at higher temperatures, and high temperature makes the rock structure expand or contract, affecting porosity and permeability.

In this study the formation temperature was estimated through the gradient method, The formation temperature at the desired depth can be calculated using the following Eq. (1):

1 $${\text{T}}_{{\text{2}}} = {\text{T}}_{{\text{1}}} + \left( {{\text{G}} \times {\text{D}}} \right)$$

where: (T_2_) is the formation temperature at the specified depth, (T_1_​) is the surface temperature, (G): is the geothermal gradient, and D is the depth^46^.

### Shale volume calculation

The volume of shale (Vsh) is a key parameter in assessing reservoir quality, as it significantly affects both hydrocarbon storage capacity and flow efficiency. Shale, primarily made up of fine-grained clay minerals, reduces the reservoir’s effective porosity. Although the total porosity may appear high, a considerable portion becomes ineffective due to the water-absorbing nature of clay and its occupation of pore spaces. Additionally, shale increases irreducible water saturation, limiting the pore space available for hydrocarbons. Permeability is also negatively impacted by shale, as clay particles obstruct pore throats, reducing pore connectivity and leading to a marked decrease in permeability^45^. Vsh can be calculated using several approaches, including linear, non-linear, neutron-density, and self-potential methods. The selection of a suitable method depends on factors such as the type of available data and the specific conditions of the reservoir^19,47^.

In the current work, the shale volume was calculated through the neutron-density method because the Nubia Formation is affected by the presence of clay minerals and K-feldspars as a cementation material which is effect on the gamma ray readings (giving higher values). Vsh was estimated from Eq. (2):

2 $${\text{V}}_{\text{s}\text{h}\text{a}\text{l}\text{e}}= \frac{({{\upvarphi }}_{\text{N}}\text{}-{{\upvarphi }}_{\text{D}}\text{}\text{})}{\left( {{\upvarphi }}_{\text{N} \text{s}\text{h}\text{a}\text{l}\text{e} }-{{\upvarphi }}_{\text{D} \text{s}\text{h}\text{a}\text{l}\text{e}}\right)\text{}}$$

where: (V_shale_) is the shale volume, (ϕ_N_) is the neutron porosity reading for the formation, (ϕ_D_) ​is the density porosity reading for the formation, (${\upvarphi }$_N shale_) is the average neutron porosity reading in a pure shale zone, and (${\upvarphi }$_D shale_) is the average density porosity reading in a pure shale zone^45^.

### Porosity estimation

#### Total porosity calculation

Total porosity represents the total pore volume in the formation, including both effective and ineffective (bound) porosity. The total porosity from neutron and density logs is averaged using the following Eq. (3):

3 $${{\upvarphi }}_{\text{T}}= \frac{{{\upvarphi }}_{\text{N}}+{{\upvarphi }}_{\text{D}}}{2}$$

where: (ϕ_T_) is the total porosity, (ϕ_N_) is Neutron porosity, (ϕ_D_) ​is the density porosity^45^.

#### Effective porosity calculation

Effective porosity is the portion of the pore volume that contributes to fluid flow, excluding bound water in clay or shale. To calculate effective porosity, total porosity can be adjusted by subtracting the shale volume’s contribution (Eq. 4):

4 $${{\varnothing}}_{\text{e}\text{f}\text{f}} = {{\varnothing}}_{\text{t}} - \left({\text{V}}_{\text{s}\text{h}} \text{*}{{\varnothing}}_{\text{s}\text{h} }\right)$$

where: (${{\varnothing}}_{\text{e}\text{f}\text{f}}$) is the effective porosity, (${{\varnothing}}_{\text{t}}$) is the total porosity, (${\text{V}}_{\text{s}\text{h}}$) is the shale volume and (${{\varnothing}}_{\text{s}\text{h}}$) is the porosity of shale^45^.

### Permeability calculation

Permeability is a measure of the simplicity with which fluids are transmitted within a rock body. It is related to the porosity but not always dependent upon it. In this study, the Wylie & Rose method was used to estimate the permeability. The Wylie & Rose method combines the traditional Wylie method and Rose’s method, and it is primarily used in clean formations or those with a small amount of shale^48,49^.

The Wylie & Rose Eq. (5) is as follows:

5 $$\text{k}=\text{a} \times {{\varnothing}}^{\text{n}}\times {(1-{\text{V}}_{\text{s}\text{h}})}^{\text{m}}$$

where: (k) is the permeability (in millidarcies, mD), (ϕ) is the porosity (in decimal form, e.g., 0.25 for 25% porosity), (a) is the tortuosity factor, (n) is the saturation factor, (V_sh_) is the shale volume, and (m) is the cementation factor^48,49^.

### Fluid saturation

#### Water saturation calculation

Water saturation (Sw​) is a crucial parameter in reservoir characterization, as it represents the fraction of the pore volume in a reservoir rock that is filled with water. Accurate estimation of water saturation is essential for determining the volume of hydrocarbons in place and for planning reservoir management strategies. Water saturation can be calculated through more than technique, in this work, water saturation was determined through two procedures which are the Pickett plot procedure and the Indonesian method.

The Pickett Plot is a graphical method for estimating water resistivity (Rw​) and other reservoir properties, such as water saturation and porosity. It plots true resistivity (Rt​) on the y-axis against porosity (ϕ) on the x-axis on a logarithmic scale. This plot is based on Archie’s Eq. (6):

6 $${\text{R}}_{\text{t}}={{\text{S}}_{\text{W}}}^{-\text{n}}\text{*}{{\varnothing}}^{-\text{m}}\text{*}{\text{R}}_{\text{w}}$$

where: (R_W_) is the water resistivity, (S_W_) is the water saturation, (R_t_) is the deep resistivity in the uninvaded zone, (Ф) is the porosity, (m) is the cementation exponent (typically 1.8–2.0), and (n) is the saturation exponent (typically around 2)^45,50^.

Water saturation was calculated by using the Indonesian method due to the presence of clay minerals cementation within Nubia Formation, which add extra electrical conductivity, even if there is no actual shale interlayer in the formation. The Indonesian method accounts for this added conductivity from the clay minerals, making it more accurate for estimating water saturation in this case. Water saturation from Indonesian method is calculated from Eq. (7):

7 $${\text{S}}_{ \text{w}}={\left(\frac{{{\text{V}}_{\text{s}\text{h}}}^{1-\left(0.5 \text{*} {\text{V}}_{\text{s}\text{h}}\right)}}{{\left(\frac{{\text{R}}_{\text{s}\text{h}}}{{\text{R}}_{\text{t}}}\right)}^{0.5}}+{\left(\frac{{\text{R}}_{\text{t}}}{\frac{{\text{a} \text{*} \text{R}}_{\text{w}}}{\text{m}}}\right)}^{0.5}\right)}^{\raisebox{1ex}{$-2$}\!\left/ \!\raisebox{-1ex}{$\text{n}$}\right.}$$

where: (S_w_) is the water saturation, (V_sh_) is the shale volume, (R_w_) is the resistivity of formation waters, (R_t_) is the true formation resistivity and (R_sh_) is the resistivity log reading in 100% shale. (Usually, *n* = 2, m = 2.15 and a = 0.62)^51^.

While the standard values for sandstone formations are typically m = 2, *n* = 2, and a = 1, in the present study, the applied values were based on actual laboratory measurements provided by the operating company. These measured parameters are: m = 1.98, *n* = 1.85, a = 1.0, and Rw = 0.03 ohm·m at 80 °F, reflecting the specific characteristics of the studied reservoir.

#### Hydrocarbon saturation calculation

To calculate hydrocarbon saturation (S_h_​), the formula used is based on the principle that the sum of the water saturation (S_w_​) and hydrocarbon saturation (Sh​) must equal 1 in the formation (Eq. 8).

8 $${\text{S}}_{\text{h}}=1-{\text{S}}_{\text{w}}\text{}$$

where: (S_h_​) is the Hydrocarbon saturation and (S_w_​) is the Water saturation^45^.

### Estimation of net pay thickness

Net pay thickness calculation is commonly based on applying cutoff values for parameters such as porosity, permeability, shale volume and water saturation to determine the economically productive layers in a reservoir. While there isn’t a single universal equation, the following is the standard approach used in the industry^52,53^. The net pay thickness is calculated from Eq. (9):

9 $${\text{Net}}\,{\text{Pay}}\,{\text{Thickness}} = \sum \left( {{\text{h}}_{{\text{i}}} } \right)$$

where: (h_i_) is the thickness of the layer that meets the defined cutoff criteria (porosity, permeability, water saturation, and shale volume)^52,53^, .

#### Common cutoff values

Cutoff values are essential in determining the net pay thickness in a reservoir, as they help to define the productive zones by excluding layers that do not meet certain criteria. The typical cutoff values which were used in the calculation of net pay thickness^52,53^ are summarized as following:

##### Porosity cutoff

- The minimum porosity required for a layer to store hydrocarbons.
- Typical Range: 10−20%, depending on the reservoir.

If the porosity of a layer is below this value, it’s generally excluded from the net pay.

##### Permeability cutoff

- The minimum permeability allows hydrocarbons to flow through the rocks.
- Typical range: 1–10 millidarcy (mD) for tight reservoirs and may be higher for more permeable formations.

Layers with permeability below this threshold are typically excluded from the net pay.

##### Water saturation cutoff

- The maximum allowable water saturation for a layer to be considered productive.
- Typical Range: 30−55%.

If water saturation in a layer exceeds this value, it may be excluded from net pay, as it’s likely not contributing hydrocarbons.

##### Shale volume cutoff

- The maximum shale volume allowable for a layer to be considered clean and productive.
- Typical Value: 35% or less.

Layers with shale volume higher than this cutoff may be excluded from net pay, as high shale content usually indicates non-productive zones^52,53^.

Based on the petrophysical evaluation of the studied reservoir, the applied cutoffs are as follows: the porosity ranges between a minimum of 0.10 and a maximum of 0.50, shale volume does not exceed 0.35, water saturation reaches a maximum value of 0.50, and permeability has a minimum cutoff value of 10 mD. These cutoffs were selected to effectively distinguish between reservoir and non-reservoir zones.

### Hydrocarbon reserves Estimation

The estimation of hydrocarbon reserves is a critical process, as it helps determine the volume of oil and gas that can be recovered from a reservoir. Accurate reserve estimation is vital for investment decisions, field development planning, and economic evaluation of projects.

In this study the hydrocarbon reserves were estimated using the volumetric method. The volumetric method is the simplest and most direct way to estimate hydrocarbon reserves. It involves calculating the total volume of oil or gas in place, based on geological and petrophysical data, and can be derived from the following Eq. (10):

10 $$\text{R}\text{e}\text{s}\text{e}\text{r}\text{v}\text{e}\text{s} \left(\text{O}\text{O}\text{I}\text{P}\right)=\frac{7758 \times \text{A} \times \text{h} \times {\varnothing} \times \left({\text{S}}_{\text{h}}\right)}{{\text{B}}_{\text{O}}} \left(10\right)$$

where: (OOIP) is the original hydrocarbon in place (Reserves), (A) is the Area of the reservoir (acres or square kilometers), (h) is the net pay thickness (feet or meters), (Φ) is the Porosity of the rock (fraction), (S_h_) is the Initial hydrocarbon saturation (fraction), and (B_O_)​ is the Hydrocarbon formation volume factor (for oil and gases) in barrels per stock tank barrel (bbl/STB) or cubic feet per standard cubic foot (scf/scf)^52,53^, .

### Recoverable hydrocarbon reserves

Recoverable hydrocarbon reserves refer to the volume of oil and natural gas that can be extracted from a reservoir using available technology under current economic and operational conditions. These reserves are estimated based on the reservoir’s physical properties, fluid characteristics, and extraction technologies.

According to the Society of Petroleum Engineers (SPE) and the Petroleum Resources Management System (PRMS), recoverable reserves are classified by^54^ into:

- Proved reserves: High probability of recovery (> 90%).
- Probable reserves: Medium probability of recovery (~ 50%).
- Possible reserves: Low probability of recovery (~ 10%).

The estimation of recoverable reserves is influenced by several important factors that determine how much hydrocarbons can be economically and technically extracted. These factors include the geological and reservoir properties like (porosity, hydrocarbon saturation, permeability, and reservoir pressure), production and recovery technologies, and economic and operational factors^55,56^.

Recoverable reserves (RR) are estimated using the following Eq. (11):

11 $${\text{RR}} = {\text{OHIP}}\times {\text{RF}}$$

where: (RR) is the recoverable reserves, (OHIP) is the original hydrocarbon in place (Reserves), and (RF) is the recovery factor “fraction”^52^.

The recovery factor (RF) is determined by reservoir characteristics and extraction techniques and varies between different types of reservoirs^57^.

The recovery factor depends on the type of reservoirs:

- Carbonate reservoirs: 15−45%.
- Conventional sandstone reservoirs: 25−50%.
- Unconventional reservoirs (shale & tight reservoirs): 5−15%.

The recovery factor depends on the type of hydrocarbon:

- Oil-bearing reservoirs: 10–40%.
- Gas- bearing reservoirs: 50–80% (due to lower viscosity and higher mobility of gas).
- Highly water saturated reservoirs: 5–15%.

The recovery factor values depending on the reservoir quality which is controlled by the porosity, permeability, and shale volume:

- High quality: 30–40%.
- moderate quality: 20–30%.
- low quality: 10–20%.

Based on the lithology, the fluid type, and the quality of the Nubia reservoir, the recovery factor was estimated to be approximately 30%.

## Results

This study is based on petrophysical data from four wells, which may not fully capture the lateral heterogeneity of the Nubia Formation. Several assumptions were applied in petrophysical calculations, such as using constant values for saturation exponent (n), cementation exponent (m), and porosity cutoffs, which may vary across different reservoir zones. Moreover, the estimation of permeability and hydrocarbon saturation is subject to uncertainty due to limitations in log resolution and borehole conditions. These factors may affect the accuracy of the volumetric reserve estimates and should be considered when interpreting the results.

### The lithology identification (Via Neutron-Density cross-plot)

**Fig. 4:**
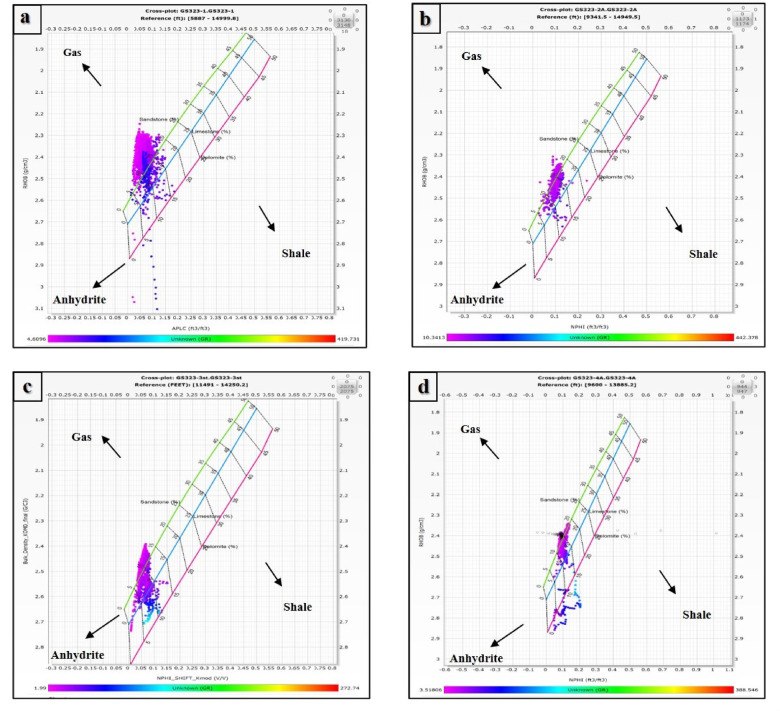
Neutron-Density (N-D) cross-plots for the Nubia Formation in the Saqqara Field, Gulf of Suez, Egypt. The plots illustrate lithological composition and fluid identification for four different wells. (**a**) N-D cross-plot of GS323-1 well, (**b**) N-D cross-plot of GS323-2 A well, (**c**) N-D cross-plot of GS323-3 well, and (**d**) N-D cross-plot of GS323-4 A well. The cross-plots highlight key reservoir components, including sandstone, shale, anhydrite, and gas-bearing zones. The clustering of data points along specific lithological trends provides insights into reservoir quality, porosity, and hydrocarbon potential. Color gradients indicate bulk density variations, with arrows marking gas, shale, and anhydrite regions. These analyses support the petrophysical assessment of the formation’s reservoir properties and hydrocarbon saturation. This figure was prepared using Techlog Software^62^.

The lithology of Nubia Formation was studied by the neutron-density cross-plots. Figure 4a–d represent the neutron-density cross-plots of the selected wells GS323-1, GS323-2 A, GS323-3, and GS323-4 A respectively for the Nubia Formation. Examining these figures, it was found that the Nubia Formation points plotted around the sandstone line indicating that the main lithology is sandstone and shifting of some points towards the anhydrite line is because of cementation.

Figure 4a shows shifting of the points also towards the gas direction (upward the plot) which indicates that the Nubia reservoir is affected by the presence of gases.

### Formation temperature determination

The geothermal gradient calculation of the Nubia Formation in the studied field ranges from 1.3 to 1.6 °FH/ 100 ft & 2.3 to 2.9 °C/100 m and formation temperature for the zone of interest is 297 FH°& 147.20 °C. Table 1 shows the geothermal calculation for Nubia sandstone reservoir.

According to^58,59^ if the formation temperature is low temperatures typically < 50 °C, that indicates immature formations for hydrocarbons, the moderate temperatures ranging from 50 to 150 °C represents the ideal range for crude oil presence, and the high temperatures (> 150 °C) may indicate dry gas presence or formations that have exceeded the oil generation stage. For the values of the geothermal gradient, the low geothermal gradient typically less than 2 °C/100 meters that indicates stable environments that are typically non-productive for hydrocarbons, the moderate geothermal gradient (2–3 °C/100 meters) represents optimal conditions for oil and gas accumulation, and the high geothermal gradient which is more than 4 °C/100 meters which suggests potential gas pockets or thermally active zones such as fault areas.

Therefore, based on the previous result for the Nubia formation, the average of the formation temperature reached 147.20 °C with an average geothermal gradient of 2.60 °C/100 m which indicates suitable conditions for the presence of heavy oil or associated gas.

**Table 1:** Combined summary of formation temperature and geothermal gradient for the Nubia reservoir in the Saqqara field, Gulf of suez.

Well name	Top Depth	Bottom Depth	X_2_-X_1_	Top Temp.	Bottom Temp.	T_2_-T_1_	gG/ft	gG/100m	gG/100ft
	(m)	(m)	(m)	(⁰C)	(⁰C)	(⁰C)	(⁰C/m)	(⁰C/100m)	(⁰FH/100ft)
GS323-1	4332.06	4543.04	211	139.8	144.8	5.05	0.024	2.39	1.31
GS323-2 A	4365.40	4556.48	191.1	140.0	145.4	5.47	0.0286	2.86	1.57
GS323-3	4160.52	4343.27	182.8	146.5	150.7	4.23	0.0231	2.31	1.27
GS323-4 A	4142.20	4228.09	85.89	153.8	156.2	2.43	0.0283	2.83	1.55

The table includes depth intervals, top and bottom formation temperatures, and geothermal gradients in both imperial (°F/100 ft) and metric (°C/100 m) units, providing a comprehensive thermal profile for each well.

### Shale volume calculation

**Fig. 5:**
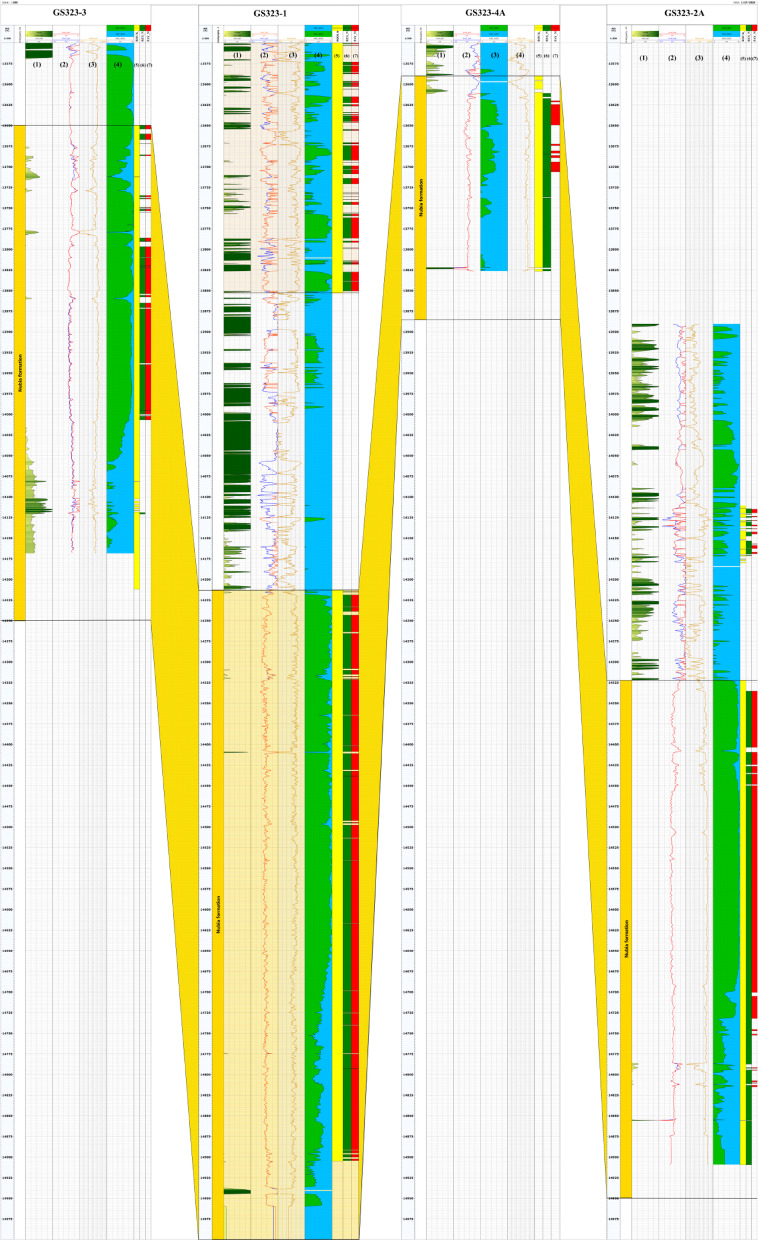
Correlation of petrophysical properties across multiple wells in the Nubia Formation, presented through a cross-section (A–B) covering wells GS323-3, GS323-1, GS323-4 A, and GS323-2 A. The figure displays various log tracks, including shale volume (“track 1”), porosities (“track 2”), where the blue curve represents total porosity and the red curve represents effective porosity, and permeability (“track 3”). Fluid saturation (“track 4”) is shown with blue shading for water saturation and green shading for hydrocarbon saturation. Additionally, rock flag indicators (“track 5”), reservoir flag indicators (“track 6”), and net pay flag indicators (“track 7”) are highlighted to assess reservoir quality. The yellow shading emphasizes key reservoir zones across the wells, illustrating lateral variations in lithology and fluid distribution. This cross-section provides insights into the continuity and heterogeneity of the Nubia Formation, aiding in hydrocarbon evaluation and reservoir characterization. This figure was prepared using Techlog Software^62^.

Based on the N-D method on the Nubia reservoir, the shale volume (Vsh) analysis in GS323-1, GS323-2 A, GS323-3 and GS323-4 A wells show a very low shale volume with average values of 0.1%, 0.1%, 2.2%, and 0% respectively, the maximum values reach 0.1% in some intervals within the studied reservoir (Fig. 5, Track 1).

Almost there is a negligible amount of shale volume within the Nubia Formation, and this very low percentage of shale volume is because of the clay minerals in the cementation materials of the Nubia sandstones, even so, it means that porosity and permeability are high, allowing fluids to move freely. This type of reservoir is generally considered high-quality and suitable for production.

### Porosity estimation

#### Total porosity calculation

Based on the application of N-D method to the Nubia reservoir wells, the average, minimum, and maximum values of the calculated total porosity (Ø _N−D_) of Nubia sandstone for each well are summarized in Table 2.

#### Effective porosity (Øeff) determination

Average, minimum, and maximum values of the calculated effective porosity (Ø_eff_) of Nubia sandstones for each well are summarized in Table 2.

According to^45,55,60,61^rocks are classified based on their porosity into different categories. Rocks with porosity greater than 20% are considered highly porous and have a high storage capacity for fluids, making them excellent reservoirs for petroleum and groundwater. These rocks can efficiently contain and transmit fluids, facilitating production. Rocks with porosity ranging from 10 to 20% are classified as good reservoirs, offering moderate storage capacity. However, they may require secondary recovery techniques, such as water or gas injections, to enhance production. In contrast, rocks with porosity between 5% and 10% are classified as low-porosity rocks, indicating limited fluid storage capacity and generally poor permeability, and enhanced recovery techniques, such as hydraulic fracturing, are often necessary to improve fluid flow. Whereas negligible or non-effective porosity is less than 5%, which represents dense rocks, such as highly compacted limestone or sedimentary rocks, where porosity is ineffective in storing or transmitting fluids.

**Table 2:** Combined summary of total and effective porosity for the Nubia reservoir.

Well name	Total Porosity calculations			Effective Porosity calculations		
	Average (Ø _*N*−D_)	Minimum (Ø _*N*−D_)	Maximum (Ø _*N*−D_)	Average (Ø_eff_)	Minimum (Ø_eff_)	Maximum (Ø_eff_)
GS323-1	14%	4.8%	16.3%	13.9%	2.2%	16%
GS323-2 A	14.8%	8%	17.2%	14.8%	4%	16.1%
GS323-3	11.5%	0.3%	12.1%	11.1%	0.1%	12%
GS323-4 A	13.7%	0.9%	15%	13.5%	0%	14.5%

The table presents average, minimum, and maximum values of both total and effective porosity across the studied wells, reflecting variations in reservoir storage capacity and fluid flow potential.

For the Nubia reservoir, the average total porosity (Fig. 5, Track 2, blue curve) across the four wells analyzed in this study is approximately 13.50%, while the average effective porosity (Fig. 5, Track 2, red curve) is around 13.35%. This indicates a minimal porosity reduction of only about 0.15%, suggesting that all the pores within the Nubia reservoir are well-connected. This finding supports the conclusion that the shale content in the Nubia reservoir is practically negligible (average ≈ 0.6%). With this porosity value, the reservoir is classified as having moderate porosity, which means that a relatively large number of pores exist between the grains, enabling the storage of significant amounts of fluids. Such reservoir characteristics may be considered favorable for commercial production and indicate substantial hydrocarbon potential, considering other petrophysical properties such as permeability and hydrocarbon saturation.

### Permeability (K) determination

The permeability of Nubia reservoir was calculated using Wyllie and Rose Equation. Average, minimum, and maximum values of the calculated permeability (K) of Nubia sandstones for each well are summarized in Table 3.

**Table 3:** Estimated permeability (K) values in millidarcies (mD) for the Nubia reservoir in Saqqara field wells (GS323-1, GS323-2 A, GS323-3, and GS323-4 A), showing average, minimum, and maximum values.

Well name	Average (K)	Minimum (K)	Maximum (K)
GS323-1	90.28 mD	0.01 mD	472 mD
GS323-2 A	355.2 mD	0.01 mD	664 mD
GS323-3	117.9 mD	0.01 mD	170 mD
GS323-4 A	524.19 mD	0.01 mD	906 mD

Permeability ranges from 0.01 to 906 mD, reflecting reservoir heterogeneity and flow potential.

According to^45,55^rocks are classified based on their permeability into different categories. Rocks with permeability greater than 1000 mD are considered high-permeability rocks, indicating excellent fluid flow capacity, commonly found in coarse-grained sandstones and fractured carbonate reservoirs. These rocks allow high production rates with minimal need for artificial stimulation. Rocks with permeability ranging from 100 mD to 1000 mD are classified as having moderate permeability, representing good reservoir quality with adequate fluid flow capacity, such as fine-grained sandstones and moderately fractured limestones. Some production enhancement techniques, such as water flooding or acid stimulation, may be required to improve recovery. Low permeability, typically ranging from 1 to 100 mD, indicates poor fluid flow capacity, often necessitating hydraulic fracturing or other stimulation techniques to enhance fluid movement. This category includes compacted sandstones and dense carbonates. Finally, non-effective permeability, which is less than 1 mD, refers to rocks with negligible permeability, such as shales and granites, where fluid movement is nearly impossible without significant artificial stimulation.

For the Nubia formation, the varying permeability values (Fig. 5, Track 3) across the four wells indicate a noticeable variation in rock properties across the sandstone reservoir. The difference in values suggests that the reservoir contains both high-permeability and low-permeability zones. High-permeability areas, such as 524.19 mD and 355.2 mD, are likely composed of coarse-grained or more porous sandstones, allowing for easier fluid flow. In contrast, areas with lower permeability, such as 90.28 mD and 117.9 mD, may be denser or have finer-grained particles, reducing the ability of the reservoir to transmit fluids. This variation may be due to geological differences or local factors such as cementation or pressure effects. Such permeability differences could lead to varying production rates from the wells, with low-permeability wells potentially requiring enhanced recovery techniques like hydraulic fracturing.

Core-derived petrophysical data, including porosity and permeability measurements, were used to validate and calibrate log-derived results. These data were obtained from GS323-2 A well over the depth interval [e.g., 14425–14825 ft]. No detailed lithological core description was available, and the analysis focused on petrophysical calibration purposes only.

**Fig. 6:**
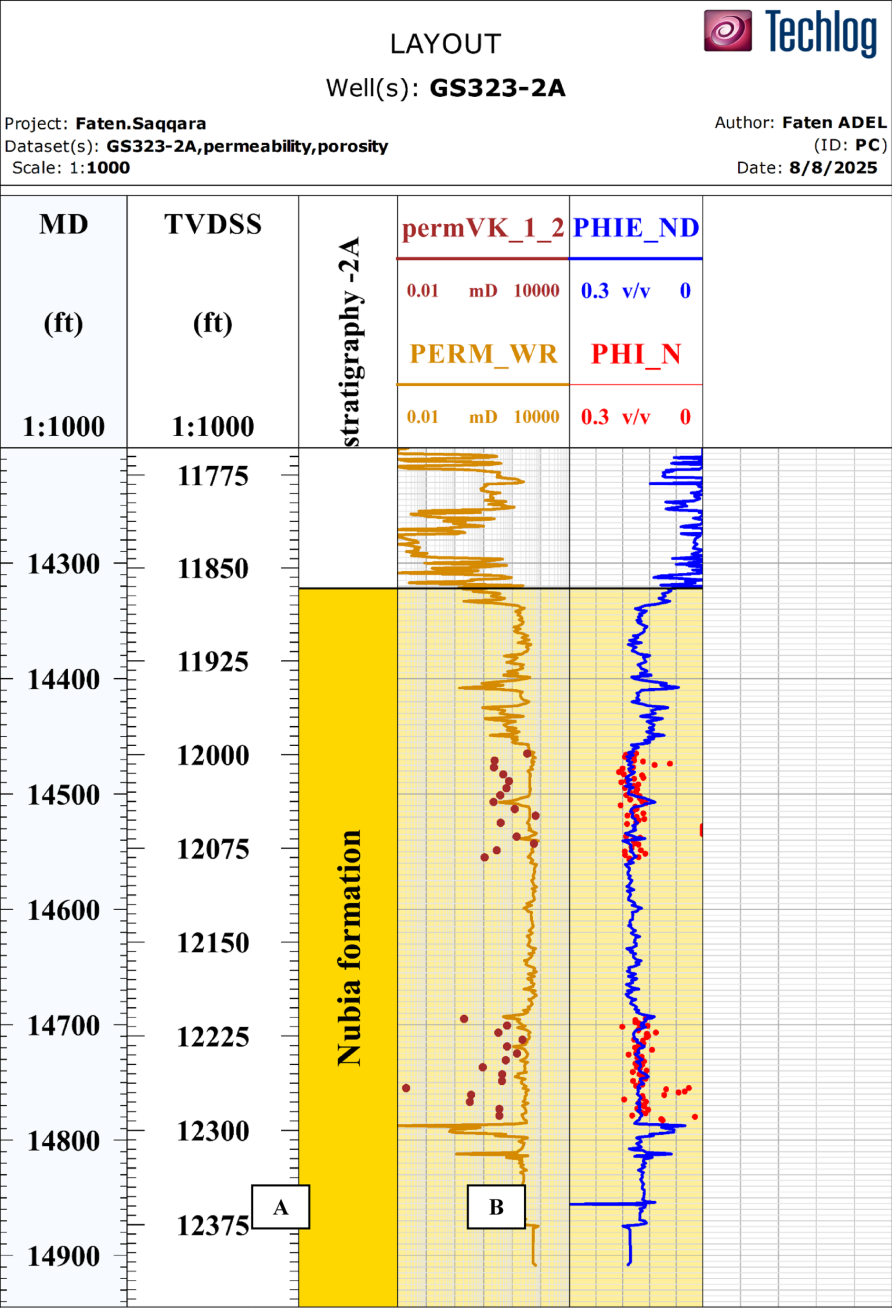
Overlay shows the calibration between the estimated effective porosity and permeability from the well logging data and the core data of the Nubia sandstones in GS323-2Awell. (A) For the effective porosity calibration and (B) for the permeability calibration.

So, Due to the availability of core data for the effective porosity and the permeability of the GS323-2 A well in the Nubia Formation, the estimated effective porosity and permeability from the well logging data was calibrated against the estimated effective porosity and permeability from the core data ss illustrated in Fig. 6 and both exhibit a good consistent in their values.

Figure 6A shows good matching between the effective porosity log from well logging data (blue curve) and the estimated core porosity log (red dots).

Figure 6B shows a good matching between the estimated permeability from well logging data (brown curve) and the estimated permeability from core data (dark red dots).

### Fluid saturation calculation

#### Water saturation (Sw) determination

**Fig. 7:**
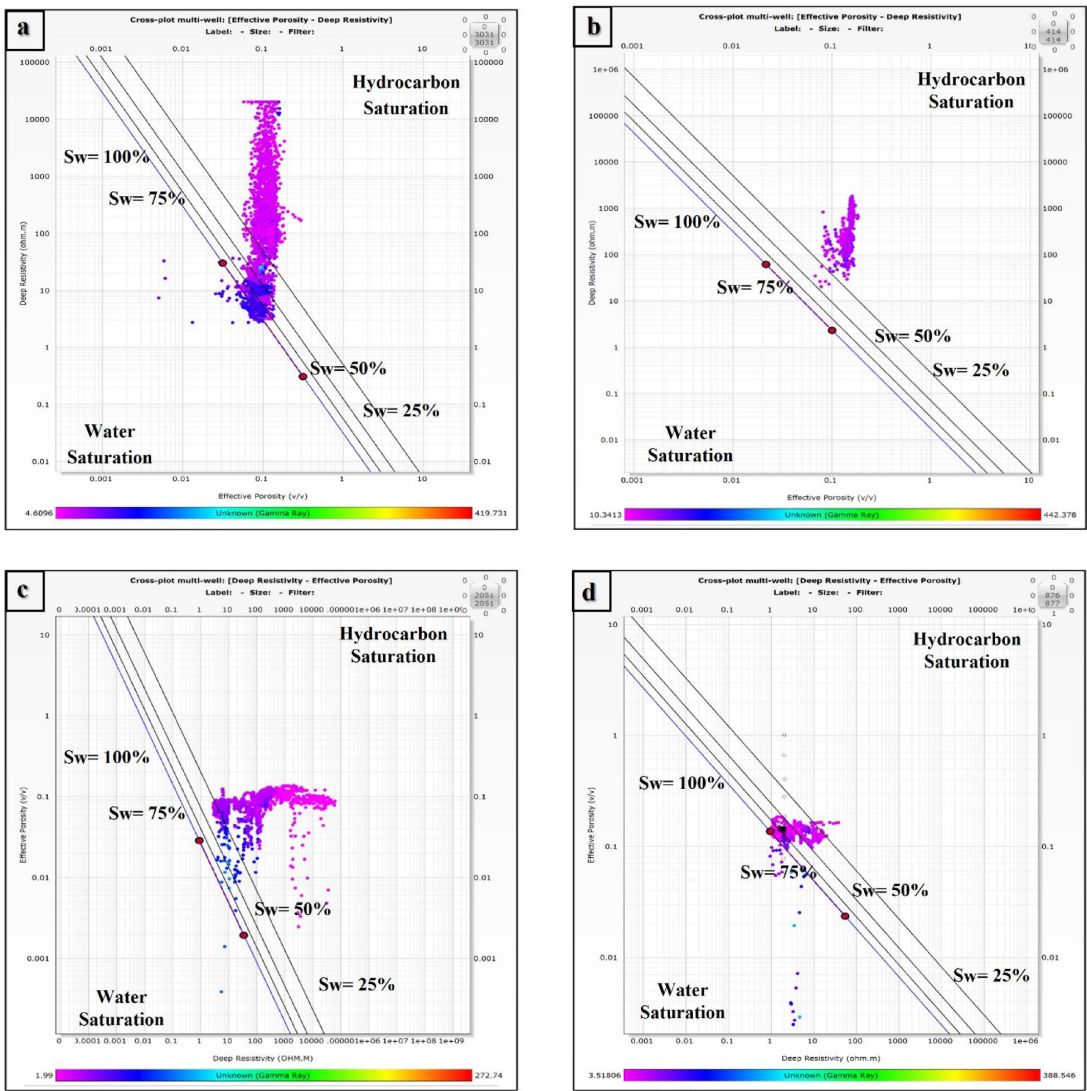
Picket plots for the Nubia Formation, illustrating the relationship between deep resistivity and effective porosity to estimate water saturation (Sw) and hydrocarbon saturation for different wells. (**a**) Represents the Picket plot for well GS323-1, (**b**) represents the Picket plot for well GS323-2 A, (**c**) represents the Picket plot for well GS323-3, and (**d**) represents the Picket plot for well GS323-4 A. The diagonal trend lines correspond to varying water saturation levels (Sw = 100%, 75%, 50%, and 25%), with data points plotted to indicate the reservoir characteristics. The hydrocarbon-bearing zones are identified towards the lower Sw regions, while the higher Sw values indicate water saturation zones. The color scale at the bottom of each plot represents the range of effective porosity, aiding in the interpretation of reservoir quality and fluid distribution. This figure was prepared using Techlog Software^62^.

The water saturation of the Nubia formation was determined via two techniques, from graphical method (Pickett plot) and mathematical method (Indonesian method). Figure 7a–c) represent the Pickett plots of the Nubia reservoir in GS323-1, GS323-2 A, and GS323-3 wells respectively, which show that the majority of the Nubia Formation points are plotted above the 25% water saturation line indicating the high hydrocarbon saturation, while Fig. 7d shows that the Nubia reservoir points of GS323-4 A well are plotted above the 50% water saturation line.

Based on the application of the Indonesian method, the average water saturation (S_w_) values of Nubia Formation are 18%, 17.5%, 9.3%, and 41% at GS323-1, GS323-2 A, GS323-3 and GS323-4 A wells respectively. It reaches 100% in some intervals, especially in the lower part of the formation at GS323-4 A well (Fig. 5, Track 4, blue filling).

Low water saturation that is denoted in GS323-1, GS323-2 A, and GS323-3 wells means a significant portion of the pore space is occupied by oil or gas rather than water. This indicates better production potentiality, as more of the reservoir’s capacity is available for hydrocarbons. In contrast, the medium water saturation at GS323-4 A indicates a balanced amount of water in the reservoir. It means there is a moderate proportion of water compared to oil or gas. Extraction may require specific strategies to handle the water content and optimize production efficiency.

#### Hydrocarbon saturation

The total hydrocarbon saturation (Sh) (Fig. 5, Track 4, green filling) was calculated using Eq. (8) and it gave average values of 82%, 82.5%, 90.7%, and 59% at GS323-1, GS323-2 A, GS323-3 and GS323-4 A wells respectively. These high values of hydrocarbon saturation signified in the Nubia reservoir indicate that the reservoir has high hydrocarbon saturation, with minimal water content. This is a desirable range for commercial production, as it implies that a significant portion of the pore space is filled with hydrocarbons, providing high production potential.

### Net pay thickness determination

After applying the cutoff values to the Nubia reservoir, which include porosity greater than 10%, permeability greater than 1 millidarcy, shale volume less than 35%, and water saturation less than 55%, the net pay thickness was estimated in the studied wells. The results showed values of approximately 481.5 feet, 435.4 feet, 154.3 feet, and 108.5 feet in wells GS323-1, GS323-2 A, GS323-3, and GS323-4 A, respectively, with an overall average of about 294.9 feet. Compared to the Nubia reservoir’s average gross thickness of approximately 468.5 feet, the average net-to-gross ratio is around 63%, indicating a reservoir of moderate to good quality. A net-to-gross ratio above 50% suggests favorable reservoir characteristics, and the variability in net pay thickness across the wells reflects heterogeneity in the petrophysical properties of the reservoir. The highest net pay thickness observed in well GS323-1 suggests that this area may have the best production potential.

The net pay distribution is illustrated in Fig. 5, Tracks 5, 6, & 7, where track 5 represents the rock flag in yellow, track 6 represents the reservoir flag in green, which applies shale content and porosity cutoffs, and track 7 represents the net pay flag in red, where all four parameters (shale content, porosity, permeability, and water saturation) are considered. The summary of the previous results is listed in Table 4.

**Table 4:** Average petrophysical parameters for the Nubia reservoir in Saqqara field (Gulf of suez, Egypt) from wells GS323-1, GS323-2 A, GS323-3, and GS323-4 A.

Well name	Depth to top (ft) TVDSS	Depth to Base(ft) TVDSS	Gross thickness(ft)	Reservoir parameters					
				Net pay thickness (ft)	Net to gross ratio	Shale volume	Effective porosity	Water saturation	Hydrocarbon saturation
GS323-1	11,982	12,594	621	481.5	0.76	0.001	0.139	0.180	0.820
GS323-2 A	11,867	12,445	578	435.4	0.75	0.001	0.148	0.175	0.825
GS323-3	11,955	12,381	426	154.3	0.36	0.022	0.111	0.093	0.907
GS323-4 A	12,354	12,603	249	108.5	0.44	0.000	0.136	0.410	0.590

Data include depth (TVDSS), gross and net pay thicknesses, net-to-gross ratio, shale volume, effective porosity, water saturation, and hydrocarbon saturation. Results highlight variations in reservoir quality and hydrocarbon potential, with GS323-3 showing the highest hydrocarbon saturation (0.907).

### Hydrocarbon reserves of Nubia reservoir

Based on the application of the volumetric method on the Nubia reservoir to calculate the original hydrocarbon in place (OHIP), Eq. (10) was applied where the average net pay thickness (h) is 294.9 ft, hydrocarbon saturation (Sh) is 78.55%, the porosity (ø) is 13.35%, the reservoir area (A) is 6000 acres, and the formation hydrocarbon volume factor (B_h_) is 1.35. The original hydrocarbon in place (Reserves) is about 106 × 10^9^ Stock tank barrels.

## Discussions

High reservoir quality in certain intervals, excellent hydrocarbon saturation, and a relatively heterogeneous reservoir with primarily sandstone lithology are shown by the petrophysical characterization of the Nubia Formation in the Saqqara Field, Gulf of Suez. However, the accuracy of the volumetric estimations and reservoir modeling is impacted by several crucial assumptions and constraints, even in the face of favorable evidence.

### Lithological, formation temperature and maturity considerations

According to regional geological models of the Nubia Sandstone Group, which is defined by fluvial to shallow marine quartz arenites, the lithology of the Nubia Formation is primarily sandstone, as deduced from neutron-density cross-plots^63^. Diagenetic cementation and the presence of hydrocarbons are suggested by the slight deviations seen toward the anhydrite and gas lines in the cross-plots. This approach is consistent with research showing how diagenesis and subsequent mineralization alter reservoir quality within Nubian-type sandstones^64^.

The detection of gas in GS323-1 by an upward shift in the neutron-density cross-plot suggests the possible existence of free gas phases, which can have a big impact on production strategy and petrophysical interpretation. It is common practice to identify gas using cross-plotting techniques, however in order to increase reliability, additional tools like nuclear magnetic resonance (NMR) logging must be used for validation and be recommended for further development plans^65^.

The Nubia reservoir is located at the upper end of the oil window, possibly moving into the gas window, with an estimated average geothermal gradient of 2.6 °C/100 m and a formation temperature of roughly 147.2 °C^66^. The preservation and production of heavy hydrocarbons or wet gas are facilitated by these heat conditions, indicating a most likely mature to post-mature hydrocarbon system. According to petroleum system modeling for the Gulf of Suez, the wells’ spatial variations in geothermal gradient, which vary from 2.31 to 2.86 °C/100 m, are within the range that is favorable for the production of both oil and related gas^67^.

### Recoverable hydrocarbon reserves

The recoverable hydrocarbon reserves of the Nubia reservoir were estimated using Eq. (11), based on the estimated reservoir properties where average total porosity is13.5%, average effective porosity is ~ 13.35%, shale volume is 0.6%, permeability is ranging from 100 to 550 millidarcies, water saturation is about 21.45%, and both oil and gas are presented. Given these characteristics, the average recovery factor was determined to be approximately 30%. Consequently, the recoverable hydrocarbon reserves of the Nubia reservoir were estimated and gave a value about 31 × 10^9^ barrels.

It is important to recognize that the calculated values for the total hydrocarbon initially in place (OHIP) and recoverable reserves are subject to certain uncertainties due to variations in key petrophysical parameters. The primary factors influencing these estimates include porosity, saturation, and recovery factor. Porosity values can vary within a certain range depending on lithology and diagenesis, typically between 10% and 25%. Saturation levels, particularly water and hydrocarbon saturation, can fluctuate due to reservoir heterogeneity, often ranging from 50 to 85%. Similarly, the recovery factor, which is influenced by the extraction technique and reservoir conditions, may vary from 10 to 40%. These variations can cause a significant range in the estimated reserves. Therefore, a sensitivity analysis or uncertainty range is essential to better understand the potential variations in the reserve estimates and improve the accuracy of resource management decisions.

### The hydrocarbon volume distribution of Nubia reservoir

The product of porosity, net pay thickness, and hydrocarbon saturation for each well serves as a key indicator of the total producible hydrocarbon volume within the reservoir. In the Nubia Sandstone reservoir, the relationship between these parameters across the studied wells reveals a notable increase in hydrocarbon volume toward the eastern part of the field. This trend suggests that reservoir properties such as porosity and hydrocarbon saturation are more favorable in the eastern region, leading to higher hydrocarbon accumulation. The observed increase in hydrocarbon volume may be influenced by several geological factors, including:

- Depositional environment variations, which may have resulted in enhanced reservoir quality.
- Structural influences, such as faulting or folding, could have created favorable conditions for hydrocarbon migration and entrapment.
- Diagenetic processes, which may have improved porosity and permeability in the eastern part of the field.

**Fig. 8:**
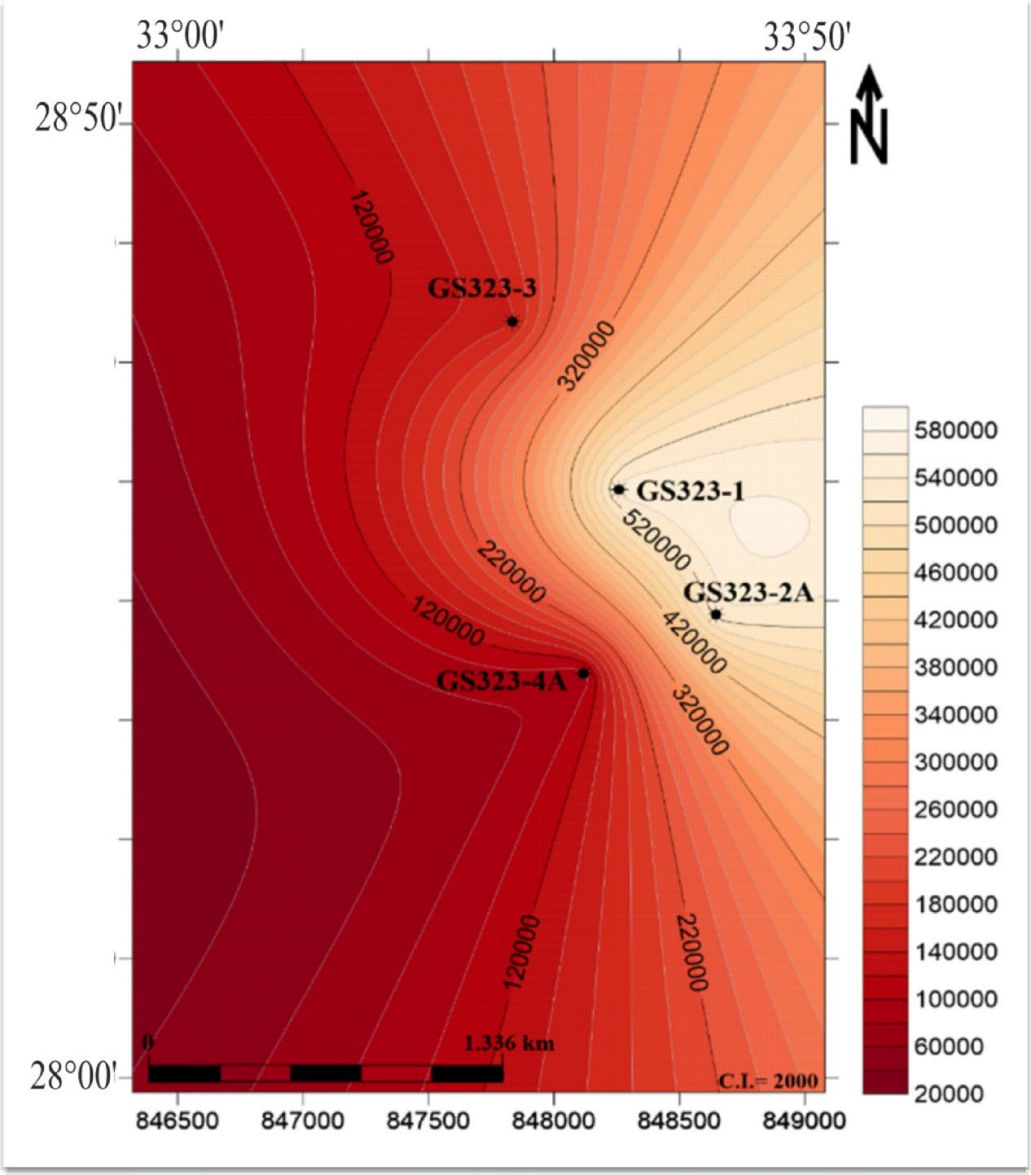
Hydrocarbon volume distribution map for the Nubia Reservoir. The map illustrates hydrocarbon volume variations derived from total porosity (Ø), hydrocarbon saturation (Shc), and net pay thickness. Contour lines and color gradients indicate hydrocarbon concentration, with lighter shades representing higher volumes near wells GS323-1 and GS323-2 A, and darker shades indicating lower volumes toward GS323-3 and GS323-4 A. This figure was prepared using Surfer Software^68^.

Understanding these controlling factors is essential for optimizing reservoir development strategies and guiding future drilling activities. As observed in Fig. 8, the hydrocarbon volume increases from the darker regions on the left toward the lighter regions on the right, with the highest accumulation occurring near the center-right portion. This area presents a promising target for hydrocarbon extraction. Based on this distribution, the study suggests drilling new wells in the eastern part of the field, where the higher hydrocarbon volume could enhance production. However, before implementing this recommendation, further validation is necessary to ensure accurate assessments and optimize drilling feasibility. To achieve this, the following steps are recommended:

- Detailed reservoir mapping to analyze the distribution of porosity, hydrocarbon saturation, and net pay thickness for precise characterization.
- Evaluation of existing production data from wells in the eastern region to confirm production trends and assess reservoir performance.
- Reservoir simulation modeling to predict the production potential of future wells and optimize drilling strategies.

By integrating these approaches, the feasibility of new well placements can be enhanced, leading to more efficient hydrocarbon recovery.

**Fig. 9:**
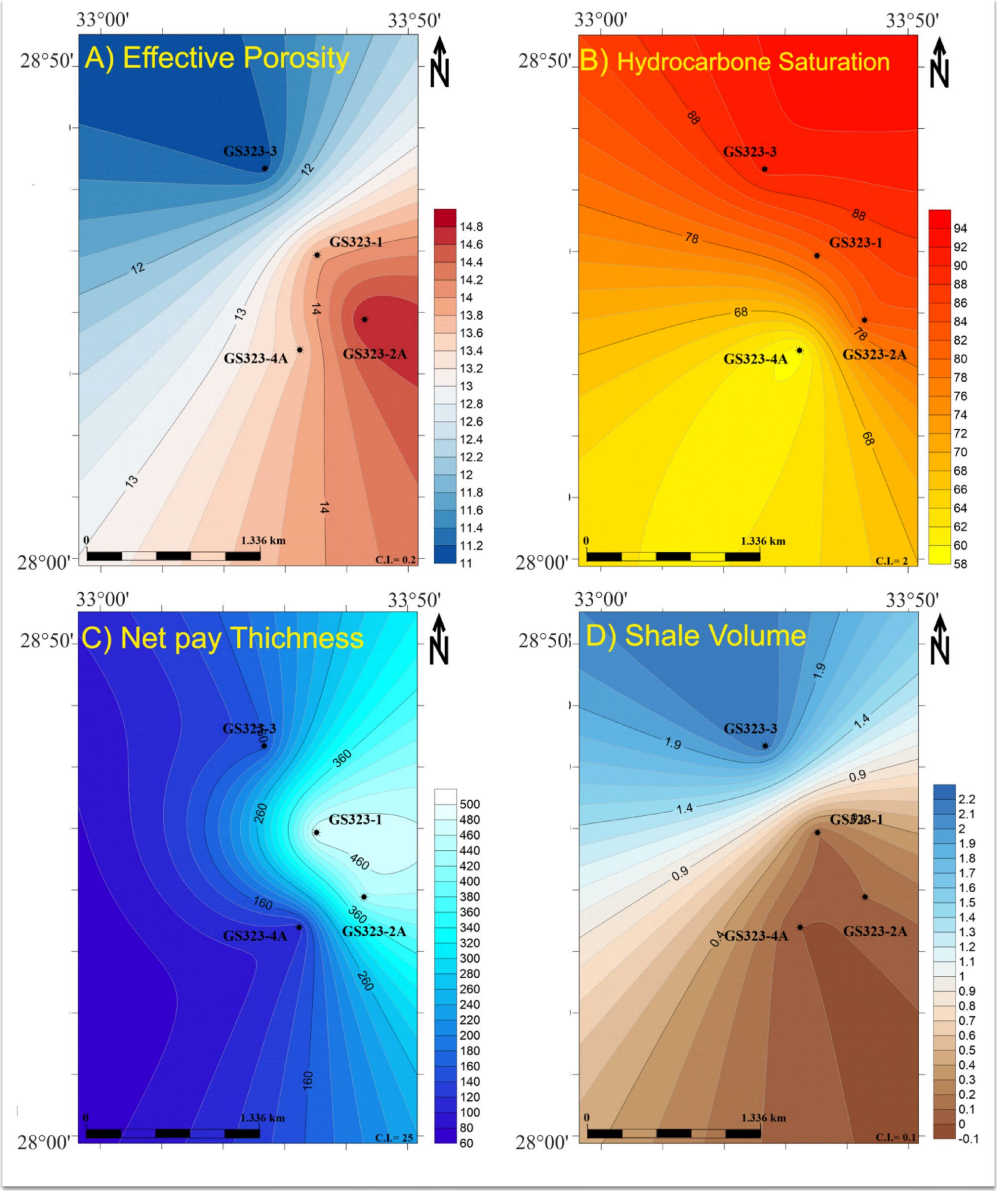
Integrated reservoir quality map for the Nubia Formation in Saqqara Field. The figure combines effective porosity (**A**), hydrocarbon saturation (**B**), net pay thickness (**C**) and Shale volume (**D**) to visualize the lateral variations in reservoir quality across the study area. Areas with higher porosity and hydrocarbon saturation and thicker net pay zones appear more favorable for production. This figure was prepared using Surfer Software^68^.

The integrated reservoir quality map of the Nubia Formation in the Saqqara Field (Fig. 9) highlights critical heterogeneities in rock properties across the study area. The map combines four key parameters: effective porosity (Panel A), hydrocarbon saturation (Panel B), net pay thickness (Panel C), and shale volume (Panel D). Regions exhibiting high porosity (> 13%) coupled with elevated hydrocarbon saturation (> 60%) and thicker net pay zones (> 90 m) are identified as prime targets for production (e.g., central and northeastern sectors). Panel D shows the distribution of shale volume within the Nubia reservoir through the studied wells, revealing that the shale content is low to nearly absent, due to the Nubia Formation lacking shales.

### Petrophysical quality: porosity, permeability, and shale volume

The shale volume is almost insignificant in all of the wells under study (average ~ 0.6%), indicating minimal clay-bound water and high reservoir cleanliness. High permeability and low capillary pressure are frequently associated with such low Vsh values, which improve hydrocarbon mobility^69^. The average total porosity and effective porosity values of approximately 13.5% and 13.35%, respectively, indicate little irreducible water saturation and well-connected pore networks. The conclusion of high reservoir efficiency is supported by this minor fall in porosity.

The Nubia reservoir is classified as a moderately porous reservoir that is appropriate for primary recovery techniques by petrophysical classification frameworks^55^. Additionally, a highly heterogeneous system is indicated by the permeability values, which vary from 90.28 to 524.19 mD across wells. Parts of the reservoir are classified as high-permeability zones due to the maximum permeability found in GS323-4 A (906 mD), which allows for outstanding flow potential and supports primary recovery without requiring a lot of stimulation^70^. Confidence in the upscaled reservoir attributes is increased and the validity of the petrophysical models is confirmed by the correlation between core and log-derived permeability and porosity in GS323-2 A.

### Fluid saturation and hydrocarbon distribution

Three of the four wells have significant hydrocarbon saturation, especially GS323-3 (90.7%) and GS323-2 A (82.5%), which is suggestive of zones with substantial recoverable reserves. On the other hand, GS323-4 A exhibits a higher water saturation (41%), which could indicate either a lower structural location within the field or possible water invasion. The confidence in Sw and Sh values is increased by using two saturation estimation techniques (Pickett and Indonesian), which is in line with industry best practices for reliable saturation determination^71^.

As is common for fluvial-deltaic sandstones such as the Nubia Formation, the hydrocarbon saturation trends and net-to-gross ratios (which range from 36 to 76%) demonstrate geographic variation in reservoir features. These differences highlight the need for specialized production methods and models at the local scale^72^.

### Hydrocarbon reserves and volumetric uncertainty

With an average recovery factor of 30% and an original hydrocarbon in place (OHIP) of 106 × 10^9^ STB, the volumetric estimation produces estimated recoverable reserves of about 31 × 10^9^ STB. Although these numbers indicate significant promise, they should be evaluated with caution because of several inherent uncertainties:

- Reservoir Heterogeneity: Because the information is based on just four wells, it does not fully capture differences in fluid distribution and lateral lithology.
- Parameter Assumptions: The saturation exponent (n), cementation exponent (m), and porosity threshold were considered to be constant values, which may not accurately represent the actual field variability^73^.
- Log Resolution and Borehole Conditions: Systematic biases in porosity and saturation computations may be introduced by limitations in tool resolution and borehole quality, especially in deviated or rugose Sect.^74^.

Incorporating core-based sedimentological research, pressure-transient testing, and seismic attribute analysis should reduce these uncertainties by improving geographical resolution and revealing reservoir connectedness^75^.

### Implications for field development and exploration

Important information for current development and future research is revealed by the petrophysical assessment of the Nubia Formation in the Saqqara Field. All of the results point to a high-quality reservoir with diverse flow characteristics, including the high average hydrocarbon saturation (up to 90.7% in GS323-3), low average shale content (< 0.6%), moderate porosity (~ 13.5%), and notable permeability variations (ranging up to 906 mD). These features suggest that field development should be both zonally and vertically stratified, and they also demand for customized reservoir management measures.

Differential production potential is indicated by the permeability variations, especially between wells GS323-4 A (average K = 524.2 mD) and GS323-3 (average K = 117.9 mD). This should be addressed by selective completion and stimulation approaches. While low-permeability segments could profit from improved recovery methods like acid stimulation or hydraulic fracturing, high-permeability zones are appropriate for primary recovery^55^. Additionally, the high degree of agreement between core and log-derived permeability and porosity in GS323-2 A supports more effective field-wide modelling by confirming the validity of wireline log interpretation for projecting reservoir parameters across unsampled intervals.

According to^66,76^the formation temperature (~ 147 °C) and the favourable geothermal gradient (~ 2.6 °C/100 m) show thermal maturity that is advantageous for the production of oil and related gas. For the purpose of directing the thermal modelling of source-reservoir systems in undrilled regions, this confirms the Nubia reservoir’s classification within the peak oil window. Together with significant net pay thicknesses (up to 481.5 feet in GS323-1) and hydrocarbon saturations, this suggests that the field will be commercially viable in both the central and northern sectors.

Although the reservoir’s clean sandstone character and small shale volume suggest lateral continuity of favourable facies from an exploration perspective, the lateral heterogeneity in porosity and permeability emphasizes how crucial it is to incorporate seismic characteristics and sedimentological modelling when placing future wells. Similar petrophysical activity may be shown by comparable lithostratigraphic units around the Gulf of Suez, indicating that this study can be used as a prediction framework to target the Nubia Formation in nearby blocks.

Furthermore, the estimated recoverable reserves of ~ 31 × 10⁹ STB and original hydrocarbon in place (OHIP) of ~ 106 × 10⁹ STB offer a solid foundation for infrastructure development and economic assessments. According to^72^these reserve amounts justify further investment for development drilling and secondary recovery systems, which may include gas injection or water flooding plans customized for the reservoir’s fluid characteristics and heterogeneity.

Ultimately, these results highlight the significant but erratic potential of the Nubia Formation. To maximize hydrocarbon recovery while lowering operational risk, optimal field development will necessitate an integrated reservoir management strategy that includes dynamic simulation, petrophysical refinement, and thorough geology modelling.

## Conclusions

This study focused on estimating the recoverable hydrocarbon reserves of the Nubia Formation in the Saqqara Field, Gulf of Suez, Egypt, through an integrated petrophysical evaluation using advanced well log interpretation techniques. The findings offer significant contributions to the global understanding of reservoir behavior in fluvio-deltaic sandstone formations, especially within mature petroleum basins. The key findings are as follows:

- Lithological analyses indicate that the Nubia Formation consists predominantly of sandstone with minor clay and anhydrite cementation. This composition supports favorable reservoir properties, offering substantial porosity and permeability conducive to hydrocarbon accumulation.
- The geothermal gradient of approximately 2.75 °C/100 m and a formation temperature of ~ 147.20 °C suggest moderate thermal maturity, reinforcing the potential for active hydrocarbon generation. Shale volume analysis confirms excellent reservoir quality, with an average clay content of only ~ 0.6%, minimizing the negative impact on porosity and fluid flow.
- The average total porosity was found to be 13.5%, with effective porosity reaching 13.35%, classifying the formation as a moderate-porosity reservoir. Permeability values varied spatially, indicating the need for localized optimization of production strategies, particularly in zones with reduced permeability.
- Hydrocarbon saturation levels were notably high (Sh ≈ 78.55%), with low water saturation, further confirming the formation’s suitability for commercial exploitation. Net pay thickness ranged from 108.5 to 481.5 feet, averaging 294.9 feet across the studied wells, reflecting substantial productive potential.
- Using volumetric methods, the estimated hydrocarbons in place are ~ 106 × 10⁹ stock tank barrels, with approximately 31 × 10⁹ barrels considered recoverable. A trend of increasing reserves toward the eastern part of the field was also observed, suggesting a promising direction for further exploration and development.
- In conclusion, the Nubia Formation in the Saqqara Field exhibits excellent petrophysical characteristics—high hydrocarbon saturation, moderate porosity, minimal shale content, and significant net pay—which collectively support its viability as a key target for future hydrocarbon development. The methodological approach and insights derived from this study can be effectively applied to similar sandstone reservoirs worldwide, contributing to improved resource management and energy security.

## Data Availability

The data that support the findings of this study are available from the Egyptian General Petroleum Corporation (EGPC) and Gulf of Suez Petroleum Company (GUPCO), but restrictions apply to the availability of these data, which were used under license for the current study, and so are not publicly available. Data is, however, available from the corresponding authors upon reasonable request.

## Abbreviations

Ф: Porosity
ФD: Density porosity
a: Tortuosity factor
A: Reservoir area
BO: Oil formation volume factor
D: Density
EGPC: Egyptian general petroleum corporation
gG: Geothermal gradient
GUPCO: Gulf of Suez petroleum company
h: Net pay thickness
hi: Thickness of the layer that meets the defined cutoff criteria
k: Permeability
m: porosity exponent
mD: Millidarcies
N: Neutron
n: Saturation factor
OOIP: Original Oil in place
PEF: Photo electric factor
PHIE or Ф_eff_: Effective porosity
PHIT or Ф_t_: Total porosity
PRMS: Petroleum resources management system
RF: Recovery factor
RR: Recoverable reserves
R_sh_: Resistivity log reading in shaly zone
R_t_: Deep resistivity in the un-invaded zone
R_W_: Water resistivity
S_h_: Hydrocarbon saturation
SPE: Society of petroleum engineers
S_W_: Water saturation
T_1_: Formation top temperature
T_2_: Formation bottom temperature
V_sh_: Shale volume
X_1_: Formation top depth
X_2_: Formation bottom depth
ρ_b_: Bulk density
Ф_Dsh_: Density porosity of shale
Ф_N_: Neutron porosity
Ф_N−D_: Neutron -density porosity
Ф_NSh_: Neutron porosity of shale
Ф_Sh_: Porosity of shale
